# Treatment accuracy without rotational setup corrections in intracranial SRT

**DOI:** 10.1120/jacmp.v17i4.6149

**Published:** 2016-07-08

**Authors:** Eeva Boman, Mika Kapanen, Marko Laaksomaa, Hanna Mäenpää, Simo Hyödynmaa, Pirkko‐Liisa Kellokumpu‐Lehtinen

**Affiliations:** ^1^ Department of Oncology Tampere University Hospital Tampere Finland; ^2^ Department of Medical Physics Tampere University Hospital Tampere Finland; ^3^ School of Medicine, University of Tampere Tampere Finland

**Keywords:** SRT, rotational setup correction, patient positioning

## Abstract

The aim of this study was to evaluate the impact of actual rotational setup errors on dose distributions in intracranial stereotactic radiotherapy (SRT) with different alternatives for treatment position selection. A total of 38 SRT fractions from 18 patients were retrospectively evaluated with rotational setup errors obtained from actual treatments. The planning computed tomography (CT) images were rotated according to online cone‐beam CT (CBCT) images and the dose distribution was recalculated to the rotated CT images using three different patient positionings derived from: 1) an automatic 6D match neglecting rotation correction $\left({\text{Auto}}_{6D}\right)$; 2) an automatic 3D match $\left({\text{Auto}}_{3D}\right)$; and 3) a manual 3D match from actual treatment $\left({\text{Treat}}_{3D}\right)$. The mean conformity index (CI) was 0.92 for the original plans and 0.91 for the ${\text{Auto}}_{6D}$ plans. The mean CI decreased significantly (p<0.01) to 0.78 and 0.80 for the ${\text{Auto}}_{3D}$ and the ${\text{Treat}}_{3D}$ plans, respectively. The mean minimum dose of the planning target volume (PTVmin) was 91.9% of the prescribed dose for the original plans and 92.1% for the ${\text{Auto}}_{6D}$ plans, while for the ${\text{Auto}}_{3D}$ and the ${\text{Treat}}_{3D}$ plans PTVmin decreased significantly (p<0.01) to 78.9% and 80.2%, respectively. No significant differences were seen between the ${\text{Auto}}_{6D}$ and the original treatment plans in terms of the dose parameters. However, the ${\text{Auto}}_{3D}$ and the ${\text{Treat}}_{3D}$ plans were statistically significantly inferior (p<0.01) to the ${\text{Auto}}_{6D}$ and the original plans. In addition, a significant negative correlation (p<0.01,|r|>0.38) was found in the ${\text{Auto}}_{3D}$ and the ${\text{Treat}}_{3D}$ cases between the rotation error and CI, PTVmin or minimum dose of gross tumour volume. In SRT, a treatment plan of comparable quality to 6D rotation correction can be achieved by using 6D registration without a rotational correction in the selection of patient positioning. This was demonstrated for typical rotation errors seen in clinical practice.

PACS number(s): 87.55, 87.57

## I. INTRODUCTION

Brain metastases occur in 10%–30% of all cancer patients.^(1)^ Treatment options are whole brain radiotherapy, local therapies (SRT or surgery), and steroids. Several trials have shown a benefit in local control, quality of life, and even improved survival for single‐ or oligometastatic patients treated with SRT.^2^, ^3^, ^4^

In SRT, small brain lesions are treated with small GTV‐to‐PTV margins in a single or few fractions. Because of the relatively high dose per fraction (typically 6–21 Gy per fraction), steep dose gradients are required around the planning target volume (PTV) to avoid damage to surrounding organs, mainly the central nervous system.^(5)^ Target localization uncertainty should be kept very small (typically within 1 mm) in SRT to not compromise the local control of the treatment^(6)^ and to minimize the risk of injury to the surrounding brain parenchyma.^(7)^

SRT presumes adequate patient immobilization. Thermoplastic stereotactic masks are nowadays commonly used in SRT treatments. These have been validated in many studies as fulfilling the high accuracy standard of SRT.^8^, ^9^ Image‐guidance with orthogonal kV‐images,^(10)^ oblique images,^(11)^ cone‐beam computed tomography (CBCT) images,^(12)^ reflecting markers with bite blocks,^(13)^ and, more recently, patient surface matching systems^(14)^ have shown to be sufficent in patient positioning in SRT keeping the residual setup errors under the desired limits.

Intensity‐modulated radiation therapy (IMRT) and volumetric‐modulated arc therapy (VMAT) have increased the dose conformity to PTVs and decreased the dose to normal tissues when compared to traditional static beams in linear accelerator‐based treatments.^(15)^ The use of VMAT also decreases the overall treatment time, reducing the possibility of intrafraction movements by the patient. The use of flattering filter‐free (FFF) beams decreases the treatment time even further in high dose per fraction treatments.^(16)^

To further increase accuracy in cranial SRT, patient rotational setup errors can be corrected using a six degrees of freedom (6DOF) couch.^17^, ^18^, ^19^ In all of these studies, the correction of the rotational setup error increased plan quality and organ‐at‐risk (OAR) sparing. It is evident that in the patient setup, the use of the 6DOF couch improves treatment quality. However, the 6DOF couch may not be available in many cancer centers due to the high cost. Gevaert et al.^(18)^ compared the calculated dose with and without rotational corrections and concluded a loss of 5% dose coverage when rotational correction was not used, pointing to a 0.5° rotation error as a threshold angle for correction.

The aim of this study was to evaluate the added value of the use of 6DOF registration in treatment without the presence of the 6DOF couch system. For this purpose, only translational corrections were taken from the 6DOF registration and the dose was recalculated using this 6D patient positioning on rotated CT images to illustrate the effect of uncorrected actual rotations on dose distribution. The resulting dose distribution was compared to that of the original plan and to those obtained by accounting for residual position errors from the 3DOF registration and the actual treatment localized by a physician.

## II. MATERIALS AND METHODS

Eighteen single‐ or oligometastatic SRT patients with a total of 38 fractions were retrospectively evaluated in this study. The number of treated fractions ranged from a single fraction to 5 fractions, depending on the tumor size and patient‐specific details. Patients were immobilized using the BrainLab mask system (Brainlab AG, Feldkirchen, Germany). A slice thickness of 1 mm was used in the initial treatment planning CT (Philips Big Bore, Philips Medical Systems, Fitchburg, WI). 6D registration was used between the recent MRI and planning CT in Eclipse registration software (Aria 11, Varian Medical Systems, Palo Alto, CA). Treatment contouring and planning were performed with the Eclipse v.11 treatment planning system (Varian Medical Systems). GTV‐to‐PTV margins of 1–2 mm were used. The VMAT technique (Rapid Arc, Varian Medical Systems) with noncoplanar or coplanar subarcs was used in the treatment planning with 6 MV or 6 MV flattening filter‐free (6MV‐FFF) beams. High definition multileaf collimation with leaf width of 2.5 mm was used in the planning. The dose calculation was performed with a grid size of 1 mm using anisotropic analytical algorithm v.11 (Varian Medical Systems). The treatments were delivered on a TrueBeam STx 1.6 with HD‐MLC (Varian Medical Systems). Patient positioning was based on online CBCT images with a slice thickness of 2 mm.

**Figure 1:**
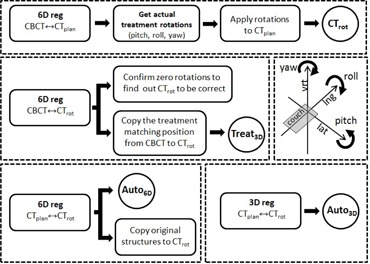
Workflow of the different image registrations and definition of rotations to construct the needed CT image set for localizations and dose calculations. The coordinate system with rotation axes is also presented.

The CBCT images were retrospectively analyzed. A schematic illustration of the workflow and the coordinate system are presented in Fig. 1. An automatic 6D registration based on skull volume was performed between the CBCT and original planning CT (CTplan) using Eclipse registration software (Varian Medical Systems). The resulting rotational deviations in all 3D directions (pitch, roll, yaw) were used in the rotation of the original CT images so that the resulting rotated CT (CTrot) presented the actual treatment orientation. The rotation isocenter was the center of the CT images. The CT rotation was done using homemade software on Matlab (The MathWorks, Inc., Natick, MA). After rotation, the new CTrot images were matched by automatic 6D registration to the CBCT image (CTrot‐CBCT 6D). No rotational deviations should be seen in this registration.

The actual treatment position $\left({\text{Treat}}_{3D}\right)$ obtained from the oncologist's 3D match was marked on the CBCT image and copied onto CTrot by using an automatic 6D registration between the CBCT and CTrot. Automatic 3D/6D registrations were used to copy the treatment position points for the ${\text{Auto}}_{3D}/{\text{Auto}}_{6D}$ plans from CTplan to CTrot, respectively. All the registrations were done using the skull as the volume of interest. The dose distributions with actual rotations were calculated in CTrot for three different positionings of the patient — ${\text{Treat}}_{3D},{\text{Auto}}_{3D}$, and ${\text{Auto}}_{6D}$ — and compared to the original treatment plan (planned).

To evaluate the target coverage and plan quality, the Paddick conformity index (CI) and gradient index (GI) were used.^20^, ^21^ CI is defined as

(1) $$CI=\frac{V100_{PTV}}{V_{PTV}}\frac{V100_{PTV}}{V100}$$

in which $V100_{PTV}$ is the prescribed dose volume in PTV, $V_{PTV}$ is the PTV volume, and *V100* is the volume which receives the prescribed dose. CI=1 means the best target coverage and lower values indicate a worse plan quality. GI is defined by

(2) $$GI=\frac{V50}{V100}$$

in which *V50* is the volume which receives higher than half of the prescribed dose. GI value increases as the dose gradient outside the target volume becomes shallower, indicating worse plan quality.

The Wilcoxon matched‐pairs, signed‐rank test (SPSS, v22, IBM Corp., Armonk, NY) for nonparametrically distributed data was performed to test the differences between the groups. Statistical significance was considered at p<0.01 (two‐tailed, |Z|>2.576) including the Bonferroni correction between the groups planned–${\text{Auto}}_{6D}$, planned–${\text{Auto}}_{3D}$, planned–${\text{Treat}}_{3D},{\text{Auto}}_{6D}-{\text{Auto}}_{3D}$, and ${\text{Auto}}_{6D}-{\text{Treat}}_{3D}$, which was made by dividing 0.05 by number of comparisons (i.e., by five).

**Figure 2:**
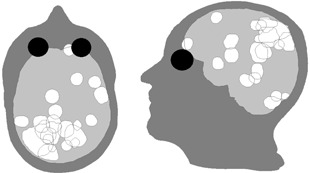
Schematic illustration of the investigated PTVs for 18 patients.

The dose distributions in four different cases (planned, ${\text{Treat}}_{3D},{\text{Auto}}_{3D}$, and ${\text{Auto}}_{6D}$) were studied for 38 different patient setups. The mean PTVvol was 8.0 cm^3^ $\left(\pm 7.6\;{\text{cm}}^{3}\right)$, with a maximum volume of 26.6 cm^3^ and a minimum volume of 0.9 cm^3^. The locations and shapes of the PTVs are presented in Fig. 2. From 38 patient setups, there were two patients with more than one lesion and treatment isocenter was located outside of the PTV. One patient had 1 fraction with two lesions and the other had 3 fractions with three lesions resulting in four different patient setups.

To demonstrate the use of 6D registration without rotational corrections for complex shaped targets or two separate spherical target volumes with reasonably large rotational errors, two patient examples are given. In the first example, the PTV is quite large (14.6 cm^3^) and complex in shape. The treatment plan consisted of two slightly noncoplanar full arcs. In the second example, two spherical PTVs were treated simultaneously using four noncoplanar half arcs. The volumes of the PTVs were 20.1 cm^3^ and 5.2 cm^3^. The distance between the PTVs was 3 cm. The GTV‐to‐PTV margin was 2 mm in both examples.

## III. RESULTS

The mean rotational setup errors were $-0.29^{\circ }\pm 0.88^{\circ }$ (range from ‐2.1° to 1.6°), $-0.26^{\circ }\pm 0.95^{\circ }$ (from ‐2.0° to 2.4°) and $-0.47^{\circ }\pm 0.80^{\circ }$ (from ‐2.7° to 0.8°) for the pitch, roll and yaw rotations, respectively. The mean distance between actual treatment positioning and the position obtained from ${\text{Auto}}_{6D}$ registration was 1.2 mm (±0.6 mm), ranging from 2.6 mm to 0.1 mm, while the corresponding distance between treatment position and that obtained from the ${\text{Auto}}_{3D}$ was 1.7 mm (±0.8 mm), altering from 3.8 mm to 0.2 mm.

CI, GI, maximum, and minimum doses of PTV and GTV (PTVmax, PTVmin, GTVmin) were investigated for each plan, and the mean, standard deviation (SD), minimum (Min), and maximum (Max) values are presented in Table 1 for each investigated group. The Wilcoxon test results for the groups planned–${\text{Auto}}_{6D}$, planned–${\text{Auto}}_{3D}$, planned–${\text{Treat}}_{3D},{\text{Auto}}_{6D}-{\text{Auto}}_{3D}$, and ${\text{Auto}}_{6D}-{\text{Treat}}_{3D}$ are shown in Table 2.

These results show that the plans from the ${\text{Auto}}_{6D}$ group have almost the same mean CI, PTVmin, and GTVmin values as the planned group and no statistically significant difference was found between these two groups. The mean CI, PTVmin, and GTVmin values were reduced significantly (p<0.01) for the ${\text{Auto}}_{3D}$ and ${\text{Treat}}_{3D}$ plans when compared to the original and ${\text{Auto}}_{6D}$ plans. Only a minor change was seen in the PTVmax among all the investigated groups. No significant difference was seen in GI values between the groups.

**Table 1:** The mean, SD, Min, and Max results for CI, GI, PTVmax, PTVmin, and GTVmin for the four different groups

*CI*	*Mean*	*SD*	*Min*	*Max*
planned	0.92	0.02	0.82	0.94
${\text{Auto}}_{6D}$	0.91	0.03	0.82	0.94
${\text{Auto}}_{3D}$	0.78	0.12	0.43	0.94
${\text{Treat}}_{3D}$	0.80	0.10	0.62	0.93
*GI*	*Mean*	*SD*	*Min*	*Max*
planned	4.23	0.83	3.14	5.93
${\text{Auto}}_{6D}$	4.23	0.82	3.14	5.91
${\text{Auto}}_{3D}$	4.24	0.83	3.14	5.94
${\text{Treat}}_{3D}$	4.22	0.83	3.12	5.92
*PTVmax*	*Mean*	*SD*	*Min*	*Max*
planned	119.8	6.5	110.6	136.5
${\text{Auto}}_{6D}$	119.8	6.4	110.6	136.4
${\text{Auto}}_{3D}$	119.5	6.4	110.5	136.6
${\text{Treat}}_{3D}$	119.9	6.6	110.6	136.8
*PTVmin*	*Mean*	*SD*	*Min*	*Max*
planned	91.9	3.1	84.9	96.4
${\text{Auto}}_{6D}$	92.1	4.0	81.8	97.5
${\text{Auto}}_{3D}$	78.9	11.7	53.7	97.0
${\text{Treat}}_{3D}$	80.2	10.7	55.1	96.1
*GTVmin*	*Mean*	*SD*	*Min*	*Max*
planned	105.5	3.6	98.2	115.6
${\text{Auto}}_{6D}$	105.7	3.4	98.4	115.6
${\text{Auto}}_{3D}$	99.9	5.4	82.7	112.8
${\text{Treat}}_{3D}$	101.1	5.7	90.2	115.1

**Table 2:** The Z‐ and p‐values from the Wilcoxon signed‐rank test for the related samples between the groups (|Z|/p)

	*CI*	*GI*	*PTVmax*	*PTVmin*	*GTVmin*
planned – ${\text{Auto}}_{6D}$	1.62/0.11	1.17/0.24	0.30/0.76	2.05/0.04	1.36/0.17
planned – ${\text{Auto}}_{3D}$	5.29/<0.01 ^a^	1.33/0.19	3.10/<0.01 ^a^	5.14/<0.01 ^a^	5.09/<0.01 ^a^
planned – ${\text{Treat}}_{3D}$	5.34/<0.01 ^a^	1.95/0.05	0.76/0.44	5.30/<0.01 ^a^	4.30/<0.01 ^a^
${\text{Auto}}_{6D}-{\text{Auto}}_{3D}$	5.29/<0.01 ^a^	2.42/0.02	3.09/<0.01 ^a^	5.30/<0.01 ^a^	5.27/<0.01 ^a^
${\text{Auto}}_{6D}-{\text{Treat}}_{3D}$	5.30/<0.01 ^a^	1.02/0.31	0.23/0.82	5.29/<0.01 ^a^	4.67/<0.01 ^a^

a ^a^ Groups with |Z|>2.567,p<0.01 were considered to have a significant difference.

**Figure 3:**
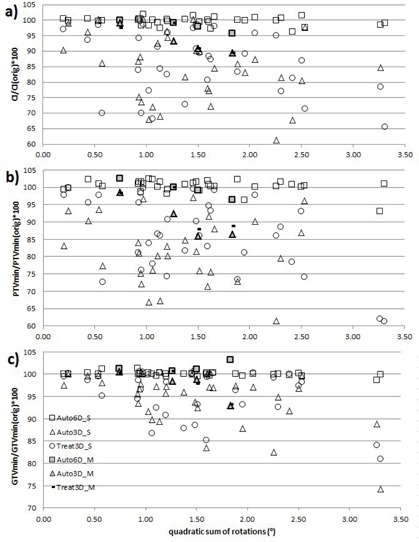
The percentage difference from the original plan to the CI, PTVmin, and GTVmin values with respect to the quadratic sum of the rotations. ${\text{Auto}}_{6D}$ plans are indicated by a square (□), ${\text{Auto}}_{3D}$ plans by triangle (Δ), and ${\text{Treat}}_{3D}$ plans by a circle (○). S indicates single lesion and M is for multiple (2–3) lesions. The multiple lesions are shown by filled symbols for ${\text{Auto}}_{6D/3D}$ plans and by a line (‐) for ${\text{Treat}}_{3D}$ plans.

Significant correlation (p<0.01) was found between the square root of the quadratic sum of the rotations (quadratic sum) and CI in the ${\text{Auto}}_{3D}$ group, $r_{\text{auto}3D}=-0.46$. In the ${\text{Treat}}_{3D}$ group, the correlation was nearly significant, $r_{\text{treat}3D}=-0.38\;(p=0.02)$. Significant correlation (p<0.01) was found between the rotation and PTVmin, $r_{\text{auto}3D}=-0.54$ and $r_{\text{treat}3D}=-0.52$, and between rotation and GTVmin, $r_{\text{auto}3D}=-0.54$ and $r_{\text{treat}3D}=-0.42$. The correlation was calculated for the percentage difference from the original plan values for the CI, PTVmin, and GTVmin values by dividing those with the original plan values to minimize the effect of variation in the original plans. No significant correlation was found in the ${\text{Auto}}_{6D}$ case between the rotation and dose parameters $\left(r_{\text{CI}}=-0.24(p=0.15),r_{\text{PTV}\text{min}}=-0.33(p=0.04),r_{\text{GTV}\text{min}}=-0.26(p=0.11)\right)$. CI, PTVmin, and GTVmin scaled to original plan values with respect to quadratic sum of the rotations are presented in Fig. 3. The multiple lesion cases are shown separately in Fig. 3. These images show a threshold of quadratic sum of the rotations of around 0.5° when all scaled parameters are under the limits of CI<90%,PTVmin<90%, and GTVmin<95% for the ${\text{Auto}}_{3D}$ group. For the quadratic sum higher than 0.5°, most of the scaled parameters are under those limits.

**Figure 4:**
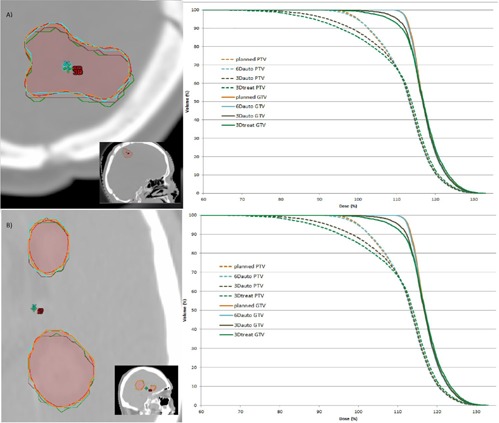
The examples of two patients (A and B) are shown with PTV outlines (red), isodoses of the prescription dose of 100% for the original plans (orange), ${\text{Auto}}_{6D}$ plans (cyan), ${\text{Auto}}_{3D}$ plans (brown), and ${\text{Treat}}_{3D}$ plans (green). The locations of the patient positionings are also shown for ${\text{Auto}}_{6D}$ (6D), ${\text{Auto}}_{3D}$ (3D), and ${\text{Treat}}_{3D}$ (green +). On the left, the DVHs are shown for each case's PTVs and GTVs.

In the first example, the PTV is nonspherical in shape, as seen in Fig. 4(a). The rotational setup errors were 0.9°, ‐1.6°, and 2.7° for the pitch, roll, and yaw rotations, respectively. The distance from the treatment positioning of the patient to the positioning given by ${\text{Auto}}_{6D}$ registration was 2.2 mm, and the distance from the treatment positioning to that given by ${\text{Auto}}_{3D}$ registration was 0.9 mm. In the second example, where there are two separate targets (Fig. 4(b)), the rotational setup errors were ‐1.8°, ‐0.3°, and 0.2°, the pitch, roll, and yaw rotations, respectively. The distance from the treatment positioning to the ${\text{Auto}}_{6D}$ positioning was 1.0 mm, and the distance from the treatment positioning to the ${\text{Auto}}_{3D}$ positioning was 0.8 mm. The isodose lines for the 100% prescription isodose and the dose‐volume histograms (DVHs) for PTV and GTV are shown in Fig. 4 for the different cases (planned, ${\text{Auto}}_{6D},{\text{Auto}}_{3D},{\text{Treat}}_{3D}$). It is evident from the DVH figures that the coverages for PTV and GTV are decreased significantly for the ${\text{Auto}}_{3D}$ and ${\text{Treat}}_{3D}$ plans, while for ${\text{Auto}}_{6D}$ the coverages are almost identical to the original plan in both examples.

## IV. DISCUSSION

6DOF couches have become a common tool in stereotactic brain treatments. Previously, the 6DOF couch has been shown to improve the treatment quality by taking into account daily rotational errors.^18^, ^19^ Common to these studies is that they compare the 6D registration with rotational correction results only to those of the 3D registration, which is also shown in this study to significantly impair the dose distribution. We have shown that by appropriate selection of the patient positioning $\left({\text{Auto}}_{6D}\right)$ in brain SRT treatments, a comparable treatment quality is achieved without the correction of rotational errors. The investigated PTVs were very different in shape, size, and location. Thus, the results give the impression that the ${\text{Auto}}_{6D}$ method works best, not only for spherical PTVs, but also for more complex PTV shapes and even for several simultaneous targets (the amount of targets varied from one to three). This is demonstrated in the examples given of nonspherical PTVs. Although there were only two cases and 4 fractions with multiple targets, their results give also an impression that the ${\text{Auto}}_{6D}$ method is the best method for patient positioning amongst the studied methods also, for multiple targets, and that at least it is better than the ${\text{Auto}}_{3D}$. However, more studies might be needed to make any conclusions for the multiple target cases.

Gevaert et al.^(18)^ proposed 0.5° as a threshold angle for the correction of rotation errors to keep the dose coverage of PTV over 95% with the 3D registration. Our data suggest that a 3D match is suboptimal in terms of CI, PTVmin, and GTVmin when the quadratic sum of the rotations exceeds 0.5° (scaled parameters CI<90%,PTVmin<90%, and GTVmin<95% in Fig. 3). For 6D registration, our results suggest that the threshold of rotations could be even larger. The quadratic sum of rotation values of 2°–3° still give adequate plan quality values for scaled CI<95%,PTVmin<95%, and GTVmin<95%. Although the quadratic sum does not describe physically the actual rotation angle, it still seems to provide reasonable one‐number measure of overall rotation.

These findings are demonstrated to be valid for brain metastases treated with the SRT. The used margins were 1–2 mm. For stereotactic radiosurgery (SRS), the clinical tolerances and practices are different and these findings may not be directly valid for SRS treatments.

Besides the setup error, the total treatment accuracy depends also on the patient intrafraction movement^(18)^ and machine isocenter accuracy. These are not considered in this work, but should be kept in mind in estimation of sufficient margins for the SRT. With mask fixation the intrafraction movement is very small, but not negligible. Verbakel et al.^(8)^ found that, for BrainLab mask system, the intrafraction motion was randomly distributed around the zero in every direction and that the maximum deviation was 1 mm and 1° when the average treatment time was 16 min. For shorter treatment times the deviation might be less.

In this paper, we have shown the 6D registration to be superior over 3D registration. However, the 6D registration may not be possible in all online registration software, and implementation in daily practice might be difficult. At least on the newest versions of Varian (Varian Medical Systems) and Elekta (Elekta Ltd., Crawley, UK) accelerators and in the ExacTrac (Brainlab) system this option is available. These results apply to Varian 6D registration and need to be confirmed before adaptation to the 6D registration systems of other vendors.

## V. CONCLUSION

We conclude that the outcome of SRT treatments without the option of 6DOF couch could be improved by using 6D registration in the treatment setup. This was demonstrated for typical actual rotation errors below 3° seen in treatment situations.

## ACKNOWLEDGMENTS

This study was financially supported by Pirkanmaa Hospital District research fund (grant number 15013).

## COPYRIGHT

This work is licensed under a Creative Commons Attribution 3.0 Unported License.
